# Application of the Electrospinning Technique in Electrochemical Biosensors: An Overview

**DOI:** 10.3390/molecules29122769

**Published:** 2024-06-11

**Authors:** Jie Liu, Zhong Dong, Ke Huan, Zhangchu He, Qixian Zhang, Dongmei Deng, Liqiang Luo

**Affiliations:** 1 School of Environmental and Chemical Engineering, Shanghai University, Shanghai 200444, China; sakura20030106@163.com; 2 College of Sciences, Shanghai University, Shanghai 200444, China; dongzhongdz@163.com (Z.D.); hk8808@shu.edu.cn (K.H.);; 3 School of Materials Science and Engineering, Shanghai University, Shanghai 200436, China; 4 Shaoxing Institute of Technology, Shanghai University, Shaoxing 312000, China

**Keywords:** electrospinning, nanofiber, morphology control, electrochemical sensors, review

## Abstract

Electrospinning is a cost-effective and flexible technology for producing nanofibers with large specific surface areas, functionalized surfaces, and stable structures. In recent years, electrospun nanofibers have attracted more and more attention in electrochemical biosensors due to their excellent morphological and structural properties. This review outlines the principle of electrospinning technology. The strategies of producing nanofibers with different diameters, morphologies, and structures are discussed to understand the regulation rules of nanofiber morphology and structure. The application of electrospun nanofibers in electrochemical biosensors is reviewed in detail. In addition, we look towards the future prospects of electrospinning technology and the challenge of scale production.

## 1. Introduction

Nanofibers, as one-dimensional nanomaterials, have attracted much attention due to their unique advantages of large specific surface areas, functionalized surfaces, and stable structures. The large specific surface areas contribute to the excellent adsorption performance of nanofibers [1,2]. In particular, nanofibers with suitable pore size distribution can provide a large number of sites to accommodate the high loading of active materials [3].

The electrospinning technique is a process of spinning polymer solutions or melts under a strong electric field. Under the action of the electric field, the spinning fluid expands into a tiny jet that solidifies into a fiber. Electrospinning devices are simple and have low cost. Electrospinning can produce a wide variety of materials, and the process is controllable. These advantages make it one of the most popular techniques for producing polymer nanofibers. In addition, carbon nanofibers can be obtained by carbonized polymer precursors [4]. Electrospinning is also a common technique for producing composite nanofibers. In contrast with the traditional spinning technique, a high-voltage electrostatic field can stretch polymer solution (or melt) into nanofiber, making the diameter of the fiber produced by electrospinning to be as small as one nanometer [5]. Moreover, the technique is simple, cost-effective, and versatile, making it suitable for industrial production, as will be discussed later in the review.

Nowadays, with the development of industry, more and more pollutants of inorganic and organic contaminants have been produced. Therefore, qualitative and quantitative analyses of these pollutants play an important role in environmental protection and food safety [6,7,8,9]. Many techniques have been developed such as fluorescence, UV-Vis spectroscopy, mass spectrometry, and electroanalysis [10,11,12,13,14]. Among them, electrochemical sensors have received more and more attention due to their excellent sensitivity, accuracy, wide detection range, easy operation, and low price [15].

In biosensors, electrospun nanofibers can be used as substrate materials or functional components of sensors. As the base material, electrospun nanofibers can provide a large surface area to enhance the adsorption of biomolecules, thus improving the sensitivity and detection limit of the sensors. At the same time, the pore structure of electrospun nanofibers is also conducive to the diffusion and transfer of biomolecules, enhancing the response speed and stability of the sensors. In addition, electrospun nanofibers can also be used as functional components, such as fixed carriers of biomolecules and fixed substrates of biometric molecules. By immobilizing biomolecules or biometric molecules onto electrospun nanofibers, a highly sensitive and selective detection of different biomolecules can be achieved.

In this review, we outline the principle of electrospinning technology, the regulation of nanofiber morphology, and the application of electrospun nanofibers in electrochemical sensors. The challenge and prospect of electrospinning technology are also prospected.

## 2. The Principle of the Electrospinning Technique

**Figure 1:**
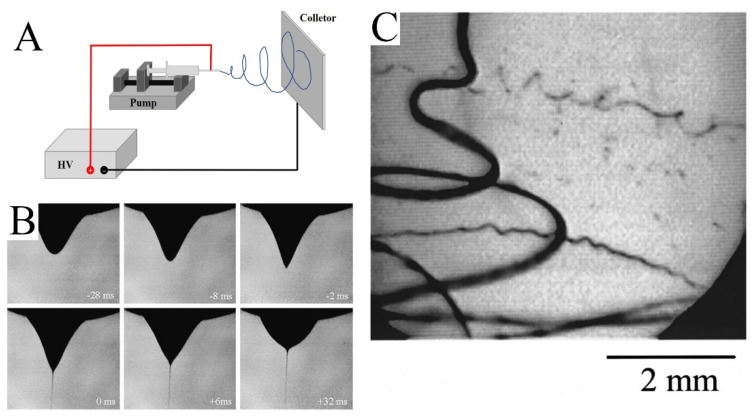
(**A**) Schematic diagram of an electrospinning device. (**B**) The photo shows the process of water droplet deformation, followed by the ejection of a jet. The time at zero was taken to be the frame in which the jet first appeared. (**C**) Image of the jet. The exposure time was 0.25 ms. (**B**) Reprinted with permission from [16]. Copyright 2008, Elsevier. (**C**) Reprinted with permission from [17]. Copyright 2000, American Institute of Physics.

As shown in Figure 1A, a simple electrospinning setup requires four basic components: a high-voltage power supply, pump, nozzle, and collector. The high-voltage power supply provides a strong electric field between the nozzle and collector. The solution is controlled by a pump and aggregates into droplets at the nozzle. Under the influence of the electric field, the shape of the droplet changes and a jet is formed (Figure 1B) [16]. Then, the jet volatilizes rapidly and tapers under the perturbation of the electric field (Figure 1C). Finally, the jet solidifies into fibers and is collected by the collector.

Organic polymers are the most often used materials for electrospinning. If organic polymers do not degrade as they dissolve or melt, they can generally be used directly for electrospinning. Depending on the functions of organic polymers, they can be divided into the following categories: (i) As the host of electrospun nanofibers: The polymers themselves have specific functions. For example, polyvinylidene difluoride (PVDF) nanofibers can be directly used in piezoelectric sensors [18,19]; (ii) As the scaffold or conducting network carrying functional materials: For example, polyacrylonitrile (PAN) nanofibers loaded with Fe_3_O_4_ nanoparticles were used for vitamin D3 detection [20]; (iii) As a sacrificial phase: For example, after the AgNO_3_/Co(Ac)_2_/polyvinylpyrrolidone (PVP) precursor nanofibers were calcined, Au-Ag/Co_3_O_4_ nanofibers were prepared, and the PVP was sacrificed in the process [21]; and (iv) As the precursor of carbon nanofibers (CNFs): Some polymers, such as PAN and PVP, were carbonized to form CNFs after thermal treatment in an inert atmosphere [22,23]. In addition, small molecules can also be used for electrospinning under special conditions, and their highly concentrated solutions or pure melts can form self-assembled structures to entangle together and behave like polymer chains [24].

In 1964, Taylor mathematically described and modeled the disintegration of drop in an electric field [25]. As reported, as the strength of the electric field exceeded the critical value, the droplets gradually changed to cones with a half angle of 49.3°. Jets were projected from these cones, which were later named as “Taylor cone”. The formation of Taylor cones is caused by the joint influence of electric field and surface tension. Liquid tends to be spherical under the influence of surface tension. The electric field causes the surface of droplets to accumulate large amounts of homogenous charges. As the electric field increases, the electrostatic repulsion on the surface of the droplet increases. When the electrostatic repulsion is stronger than the surface tension, the droplet will deform into a cone and form jets. After that, Taylor published two articles to preliminarily explore the behavior of the jet in the electric field in 1966 and 1969 [26,27].

**Figure 2:**
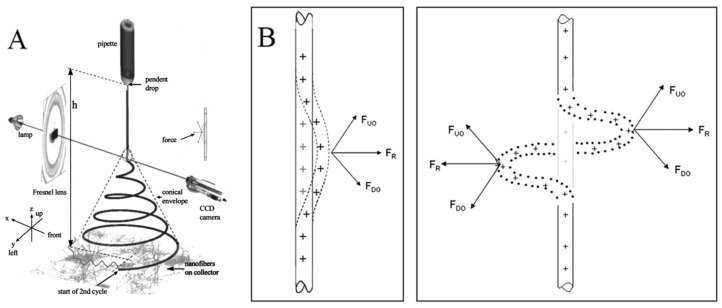
(**A**) An electrospinning jet that contained two successive electrical bending instabilities; (**B**) affected by the repulsion forces between charges, the perturbation segment of the jet (the dashed segment) is affected by the repulsion forces F_UO_ and F_DO_ of the lower and upper charges. The resultant of these forces FR is in the radial direction of the jet, which makes the jet more curved. (**A**) Reprinted with permission from [17]. Copyright 2000, American Institute of Physics. (**B**) Reprinted with permission from [16]. Copyright 2008, Elsevier.

The electrospinning method is the polymer-eruption electrostatic drawing spinning method. Firstly, the polymer solution or melt is subjected to a high-voltage electrostatic charge ranging from several thousand to ten thousand volts, and the charged polymer droplets are accelerated at the Taylor cone pole of the capillary under the effect of an electric field force. When the electric field force is large enough, the polymer droplets overcome the surface tension to form an eruptive trickle. The thin stream evaporates or solidifies in the process of eruption and eventually falls on the receiving device to form a nonwoven-like fiber felt. The dynamics of an electrically charged jet in an electric field are complex. When the jet is first formed, it is stretched along the electric field. At this segment, the jet can keep moving in a straight line. But the jet moves radially outward at a comparable velocity because of the electrical bending instability (Figure 2A). Around the 2000s, many studies attempted to build a physical or mathematical model of the jet at this segment [17,18,28,29,30,31,32]. The linear jet will bend under perturbation. The charge carried with the bend segment moves downward and outward by the repulsion (F_DO_) above the bend region, and at the same time, the charge moves upward and outward by the repulsion (F_UO_) below the bend region. The result of the two forces FR is radial and increases exponentially with the increasing degree of jet bending (Figure 2B) [16]. As a result, the jet appears as a spiral with a cone envelope. When the jet continues to move in the electric field, the jet that has been bent into a spiral shape will obtain the second bending instability, as indicated at the end of the jet in Figure 2A. The jet is stretched thinner and thinner in the process.

There are many factors affecting the preparation of nanofibers by electrospinning, which can be divided into solution properties (such as viscosity, elasticity, electrical conductivity, and surface tension), control variables (such as the static voltage in the capillary, the potential of the capillary port, and the distance between the capillary port and the collector), and environmental parameters (such as solution temperature, air humidity and temperature in the spinning environment, and airflow speed). The main influencing factors include: (1) The concentration of polymer solution: The higher the concentration of polymer solution, the greater the viscosity and the greater the external tension. The droplet cleavage can be weakened by increasing the external tension after leaving the nozzle. Generally, when other conditions remain constant, the fiber diameter increases with the increase in concentration; (2) Electric field strength: With an increase in electric field strength, the jet of the polymer electrospinning solution has a larger outer charge density and thus has a greater electrostatic repulsion. At the same time, the higher electric field strength makes the jet obtain a greater acceleration degree. Both of these factors can cause the jet and fiber to have greater tensile stress, resulting in a higher tensile strain rate, which is conducive to the preparation of finer fibers; (3) The interval between the capillary port and the collector: After the polymer droplets are ejected through the capillary port, they volatilize in the air with the solvent, and the polymer is concentrated and solidified into fibers, which are finally accepted by the receiver. As the distance between the two increases, the diameter decreases; (4) Activity rate of electrospinning fluid: When the diameter of the spinneret is fixed, the average jet velocity is obviously proportional to the diameter of the fiber; (5) The condition of the collector: The condition of the collector is different, and the condition of the produced nanofibers is also different. When the fixed collector is used, the nanofibers appear to have random irregular scenes. When using a rotating disk collector, the nanofibers appear in a parallel pattern. Therefore, the fibromomentum produced by different equipment conditions is different.

In addition to spiraling, the jet will also undergo other shape changes, such as branching and the formation of beads. The undulations increase as the charge density of the jet increases. When the undulations are large enough to become unstable, the jet will branch off. The undulations come from the combined effects of the electric Maxwell stresses and surface tension [31]. Branching occurs more frequently in viscous solutions and at high electric fields. In contrast, when the charge density of the jet is reduced, surface tension will dominate in the competitive relationship between the electric field and surface tension. Capillary instability that results in the transformation of the jet into spherical droplets occurs.

In recent years, researchers have tried to predict the diameter of electrospun nanofibers in various ways, including theoretically or experimentally. Gadkari summarized the literature on various correlations and analyzed them to obtain the relationship dependency of the nanofiber diameter on the viscous and surface charge repulsion effects [33]. A design of the experiments model, analyzing data based on polynomial equations without physical meaning, was proposed by Ruiter to identify the effects of electrospinning parameters on scaffold morphologies of poly-D,L-lactic acid [34]. The model shows that solution concentration plays an important role in the morphology of nanofibers.

## 3. Regulation of Nanofibers

Diameter, as the most basic parameter of nanofibers, has always been the object of greatest concern to researchers. A variety of investigations have been reported on the effect of electrospinning parameters on the diameter of the nanofibers, both theoretically and experimentally. The diameter of the nanofibers is influenced by the concentration of polymer in solution, the type of solvent, the conductivity, as well as the feeding rate of the solution [35,36]. The higher the concentration and viscosity of the solution, the higher the voltage required to overcome its own surface tension to form the jet; the voltage needs to be optimized to match with the feeding rate of spinning fluid and the collecting distance for producing nanofibers with a uniform diameter.

### 3.1. Diameter of Nanofibers

It has been reported that the larger the voltage, the smaller the diameter of the nanofibers [37]. However, there was also the observation that the diameter of the nanofibers increased with an increase in the voltage [38]. The reason is that the type of spinning solution and the parameters are not the same. This indicates that the influence of the voltage summarized by simple control variables on the final diameter of the nanofibers is not rigorous enough. Gu et al. reported that the concentration of the spinning fluid has a greater effect on the diameter of the nanofibers than the voltage [39]. In fact, the main factor affecting the diameter of the nanofibers here is the viscosity of the solution, which changes with the concentration of the solution and the molecular weight of the polymer [39,40].

Fridrikh et al. reported that there is a limiting diameter for the jet, which arises from a force balance between surface tension and electrostatic charge repulsion [41]. These two forces are determined by the viscosity and conductivity of the solution, respectively. In the case of ignoring the elastic effect and fluid evaporation, the diameter of the nanofibers can be given by the following equation:

(1) $$h_{t}={\left(\gamma \bar{\varepsilon }\frac{Q^{2}}{I^{2}}\frac{2}{\pi \left(2\ln\chi -3\right)}\right)}^{1/3}$$

where *γ* is surface tension of the solution, $\bar{\varepsilon }$ is the dielectric constant, *Q* is the flow rate of the solution, *I* is the current carried by the jet, and *χ* is proportional to the ratio of the jet diameter h and radius of curvature R. It is shown that the nanofiber diameter decreases with the increase in the charge carried by the jet. However, the electrospinning process is much more complex than this model, so this model is not accurate or comprehensive enough. For example, temperature is an easily overlooked parameter. Most solution electrospinning processes take place at ambient temperature. As the temperature increases, the viscosity and surface tension of solution decrease, leading to a reduction in nanofiber diameter. But at the same time, the increase in temperature will also cause the acceleration of the evaporation of the solvent. In this case, the drawing process, which is also the fining process of the nanofibers, will end prematurely. A temperature equilibrium that minimizes the diameter of the nanofibers can be found [42].

### 3.2. Morphologies and Structures of Electrospun Nanofibers

Electrospinning has become one of the main ways to prepare nanofibers because of its advantages such as a simple manufacturing device, good adjustable fiber structure, and strong technical combination. It can be used for directly and continuously spinning polyester, polyurethane, polyethylene, and other polymers into ultra-fine and uniform fibers with diameters ranging from less than 3 nm to more than 1 μm and depositing on the receiving plate to obtain non-woven fabric. It is also possible to prepare nanofibers with special shapes by electrospinning in addition to nanofibers with uniform diameters. Porous nanofibers, hollow nanofibers, and bead-on-string nanofibers are produced to meet the requirements of various applications. More complex structures tend to yield better performance, such as a greater specific surface area, rougher surface, or greater surface energy. Next, we will discuss the strategies for producing nanofibers with different morphologies and structures.

#### 3.2.1. Bead-on-String Nanofibers

**Figure 3:**
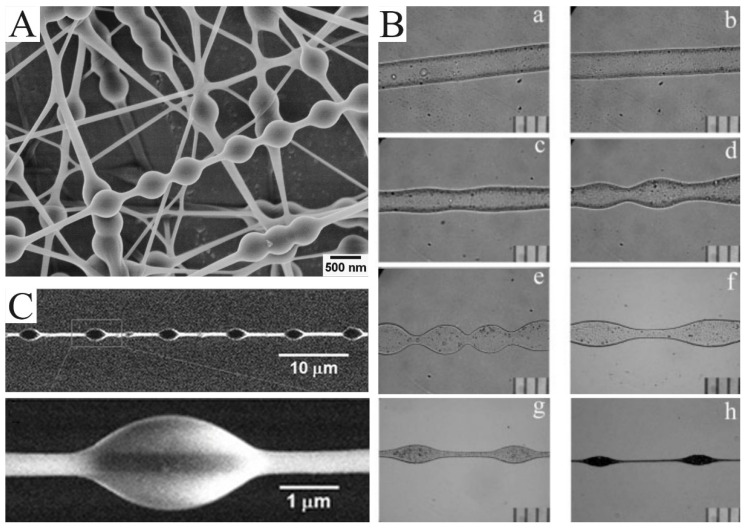
(**A**) SEM images of the bead-on-string nanofibers with polyvinyl alcohol (PVA): polystyrene (PS) (1:1) and 9 wt.% PVA for 473 nm PS nanospheres. (**B**) The bead formation process at different points along the PHBV nanofibers formed by electrospinning. The photos were taken at different distances from the needle tip according to their orders: (**a**) 1 cm; (**b**) 3 cm; (**c**) 5 cm; (**d**) 7 cm; (**e**) 9 cm; (**f**) 12 cm; (**g**) 15 cm; (**h**) 30 cm. (**C**) SEM image of PS nanofibers with a regularly distributed polyethylene glycol (PEG) droplet on it. (**A**) Reprinted with permission from [44]. Copyright 2012, American Chemical Society. (**B**) Reprinted with permission from [43]. Copyright 2005, Society of Plastics Engineers. (**C**) Reprinted with permission from [45]. Copyright 2011, WILEY-VCH.

By adjusting the parameters in the process of electrospinning, electrospun nanofibers with unique morphologies can be simply obtained. Bead-on-string nanofibers are typical. As we discussed earlier, the jets need to overcome capillary instability to form uniform nanofibers. Otherwise, the bead structure will be obtained. At first, the bead structure appears randomly, which is considered an undesirable by-product. It is not until the production of uniform bead-on-string structures that attention is paid to this structure (Figure 3A). Zuo et al. reported how and why this structure was formed [43]. As indicated in Figure 3B, the jet is photographed at different distances from the nozzle. As the jet flows further and further away from the nozzle, the smooth surface of the jet flow first becomes wavy, then dumbbell-like, and finally bead-shaped.

The bead-on-string structure can be regulated by controlling viscosity, net charge density, and surface tension [46]. Charge repulsion and electric field force are the causes of the drawing and deformation of the jet. Surface tension always makes the liquid the smallest surface area by turning into a sphere, while viscoelastic force resists rapid changes in shape. Therefore, the easiest strategy for producing bead-on-string nanofibers is adjusting the solution concentration and applying voltage. A higher concentration enhances electrostatic repulsion forces and viscoelasticity but reduces surface tension. A polymer solution with a lower concentration tends to produce beads with a higher density [47]. The effect of the viscosity of spinning solution on bead structure can be clearly reflected in an example. By combining electrospinning and electrospraying, Tian et al. obtained PEG beads on PS-string hetero-structured nanofibers in a coaxial jetting process (Figure 3C) [45]. During electrospinning, the outer PEG with a low viscosity is deformed by humidity, while the PS with a high viscosity keeps the shape of a “string”.

The surface tension and viscoelasticity of the jet can be changed by adjusting the proportion of solvent. For example, PS-b-poly(ethylene butylene)-b-PS triblock copolymer solutions, which can be dissolved in different proportions of tetrahydrofuran/N,N-dimethylformamide (THF/DMF), have different rheology data. Accordingly, the viscosity of the solution will vary considerably [48]. In addition, a corona discharge can be used to add neutralizing charge to the jet so as to reduce electrostatic repulsion [46].

#### 3.2.2. Porous Nanofibers

**Figure 4:**
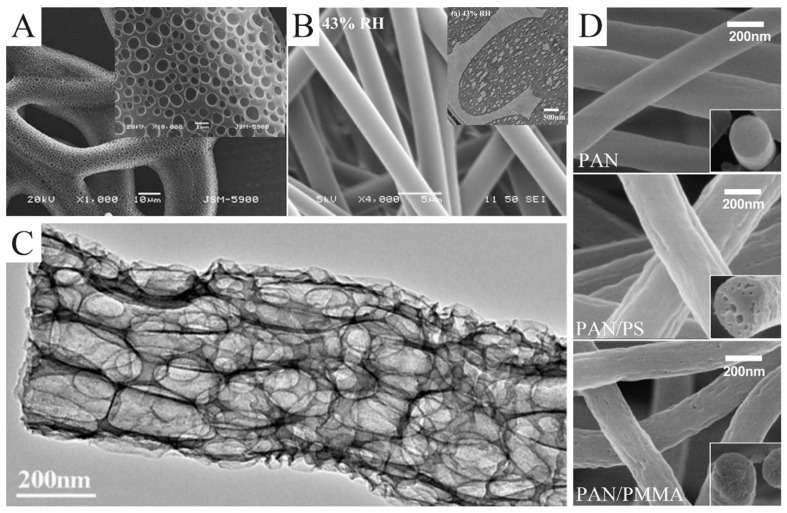
(**A**) SEM images of electrospun poly(ε-caprolactone) fibers collected in water as a function of pH 3. (**B**) SEM images of as-spun fibers electrospun from a 30 wt.% PS/DMF solution under 43% relative humidity. The illustration is the cross-sectional TEM images of them. (**C**) TEM images of the macroporous CNFs obtained by etching SiO_2_/Sb@CNF composites with an aqueous HF solution. (**D**) SEM images of the porous CNFs produced from pure PAN, PAN/PS, and PAN/PMMA, respectively. (**A**) Reprinted with permission from [61]. Copyright 2011, Springer. (**B**) Reprinted with permission from [52]. Copyright 2009, American Chemical Society. (**C**) Reprinted with permission from [62]. Copyright 2018, American Chemical Society. (**D**) Reprinted with permission from [63]. Copyright 2016, Elsevier.

In certain cases, the rapid evaporation of the solvent causes the liquidation of water vapor during the spinning process. Droplets formed by water vapor liquefaction adhere to the jet surface, leading to the formation of pores on the fiber’s surface (Figure 4A) [49]. This approach, which is essentially vapor-induced phase separation, has to meet two requirements: humid environment and volatile solvents, such as THF, methylene chloride, and carbon disulfide. The size and number of pores can be easily controlled by changing the humidity. The higher the humidity, the larger the pore size and the higher the pore density [50]. In addition to controlling environmental humidity, there are other methods of inducing phase separation. McCann et al. electrospun PAN nanofibers into liquid nitrogen [51]. In this process, pores were introduced into the nanofibers surface by thermally induced phase separation. Pores could not only be formed on the surface of the nanofibers but also inside the nanofibers so as to obtain nanofibers with a loose structure. As shown in Figure 4B, Pai et al. used PS/DMF solution to produce fibers with a smooth surface and a porous interior [52]. Compared with a highly volatile solvent mentioned earlier, the evaporation rate of DMF is not fast enough to form small droplets on the jet surface. DMF absorbed water vapor in the air due to a non-solvent phase separation reaction between them. As a result, a porous structure was formed inside the fibers. The growth process of porous structures resulting from phase separation was calculated by Dayal et al. based on the Cahn–Hilliard time-evolution equation and solvent evaporation rate equation [53]. The strategy of introducing pores through phase separation has been applied to a variety of polymers, such as PS [54], poly(L-lactic acid) [55,56], polyvinyl butyral [57], polyethylene terephthalate [58], and poly(ε-caprolactone) [59,60].

Porous nanofibers can also be produced through the removal of a sacrificial phase. Commonly, the sacrificial phases are salt, polymers, and nanoparticles. A sacrificial phase of salt can be removed by leaching. Gupta et al. used GaCl_3_ as a template to prepare porous nylon-6 fibers [64]. Wang et al. prepared SiO_2_/Sb@CNFs by carbonizing electrospun precursors, which were etched with HF solution to remove SiO_2_ and Sb nanoparticles [62]. What was left were porous CNFs (Figure 4C). The porous CNFs could also be prepared by electrospinning PAN blending with the sacrificial PS or PMMA [63]. PS and PMMA were easier to pyrolyze than PAN. As a result, PS or PAMM was completely pyrolyzed to form pores during carbonization. Moreover, PS foam (Figure 4D) and Nafion are also suitable as a sacrificial phase in PAN solution [65,66].

The introduction of pores has other advantages in addition to greatly increasing the specific surface area of the nanofibers: the functionalization inside the pores is easy to realize for obtaining functional surfaces [67]; and porous CNFs have stronger ionic adsorption and higher specific capacitance [65].

#### 3.2.3. Hollow Nanofibers

**Figure 5:**
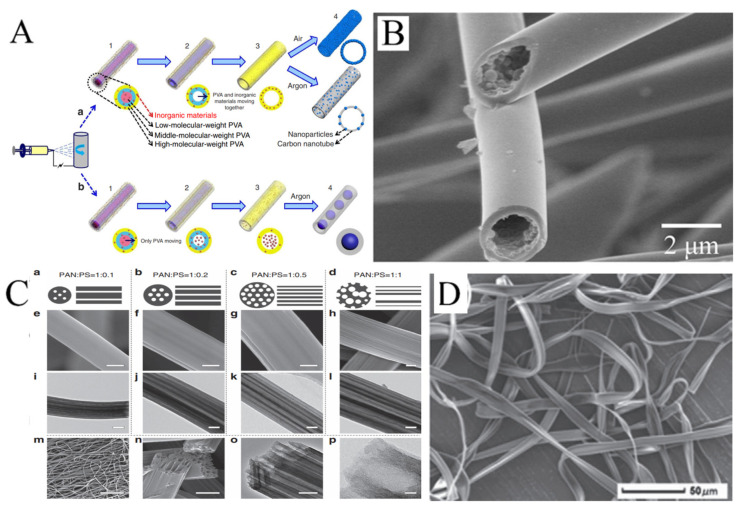
(**A**) Schematics of the gradient electrospinning and controlled pyrolysis method. (**B**) SEM images of hollow silicon carbide nanofibers. (**C**) (**a**–**d**) Schematic diagrams, (**e**–**h**,**m**,**n**) FESEM and (**i**–**l**,**o**,**p**) TEM images of LRC nanofibers based on various PAN/PS weight ratio: (**a**,**e**,**i**) 1:0.1, (**b**,**f**,**j**) 1:0.2, (**c**,**g**,**k**,**m**–**p**) 1:0.5, (**d**,**h**,**l**) 1:1. Scale bars, 200 nm (**e**–**l**,**o**), 20 μm (**m**), 500 nm (**n**), 20 nm (**p**). (**D**) SEM images of microfibers electrospun from PMAGH-block-PS-block-PMAGH in 0.20 g mL^−1^ CHCl_3_ solution at 20 µL min^−1^ feeding rate. (**A**) Reprinted with permission from [68]. Copyright 2015, Macmillan. (**B**) Reprinted with permission from [69]. Copyright 2018, Elsevier. (**C**) Reprinted with permission from [71]. Copyright 2015, WILEY-VCH. (**D**) Reprinted with permission from [72]. Copyright 2019, WILEY-VCH.

Hollow nanofibers are another common structure of electrospun nanofibers. There are two strategies for preparing hollow nanofibers. One is coaxial electrospinning, which we will discuss later. The other strategy is to make the components of the solution spontaneously move along the radial direction of the jet and be stratified by adjusting the ratio of a mixed polymer solution. Niu et al. produced hollow PVA nanofibers by mixing PVAs with different molecular weight [68]. The schematic diagram of the formation of the hollow structure is displayed in Figure 5A. With the same electrospinning parameters, the higher the molecular weight, the higher the distribution in the outer layer. The nanofibers were then carbonized at high temperatures. In this process, the low-molecular weight PVA in the inner layer first pyrolyzed and moved to the high-molecular weight PVA. Hollow CNFs were produced. It is of concern that if inorganic materials were mixed into the solution and the nanofibers carbonized in the protection of argon, the inorganic materials would not move with the low-molecular weight PVA. The inorganic materials aggregated into spheres in the channels of hollow nanofibers, and pea-like nanofibers could be formed. Hollow nanofibers are commonly prepared by similar methods. For example, the fibers have been produced by electrospinning with a mixture of polycarbosilane (PCS) and PS, with PCS in the outer layer and PS in the inner layer. After carbonization, hollow SiC nanofibers were formed, as PCS turned into SiC and PS was pyrolyzed (Figure 5B) [69]. Hollow nanofibers can also be produced based on vapor-induced phase separation. In another example, camphene and tetraethoxysilane, as pore-forming agents, could be added into PS solution. Water entered the surface of the jet in the electrospinning process. At this point, tetraethoxysilane that diffused to the outer layer evaporated, and camphene that diffused to the outer layer was removed after freeze-drying. Hollow and porous structures of electrospun PS fibrers were obtained [70].

Some hollow fibers with more interesting structures have been reported by electrospinning. For example, hollow fibers with multiple channels produced with a mixture of PAN and PS have been reported. PS as the sacrificial phase will form more than one channel inside PAN. The number and distribution of channels will vary with the ratio of PAN to PS in the solution (Figure 5C) [71]. In addition, it is possible to obtain ribbon fibers in the production of hollow fibers, and unique polymer skin can form on the surface of the jet. After that, the evaporation of solvent will result in the vacuum inside, leading to the collapse of skin (Figure 5D) [72,73,74].

## 4. Application of Electrospun Nanofibers in Electrochemical Biosensors

Electrochemical biosensors have attracted more and more attention due to their speed, simple operation, and low cost. Electrospun nanofibers have been extensively applied in electrochemical biosensors for the determination of various molecules such as glucose, hydrogen peroxide (H_2_O_2_), uric acid (UA), dopamine (DA), ascorbic acid (AA), protein, and amino acids.

### 4.1. Glucose Sensors

As a source of energy in the body, glucose is widely found in every corner of our life. People who want to lose weight need to know the amount of glucose in their diet in order to control their diet properly. Diabetics need to regularly measure their blood sugar levels to keep themselves healthy.

**Figure 6:**
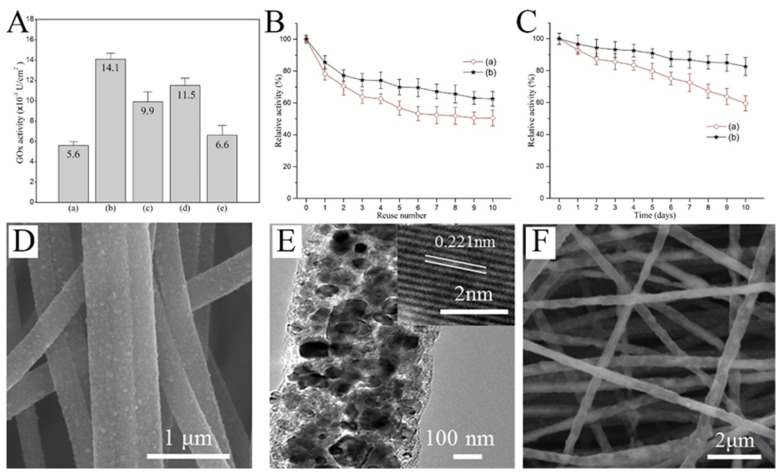
(**A**) Activity of GOx immobilized on the surfaces of (**a**) untreated and (**b**) air-, (**c**) nitrogen-, (**d**) CO_2_-, and (**e**) argon plasma-treated PVA/malonic acid nanofibers. (**B**) Reusability of GOx immobilized on (**a**) unmodified and (**b**) air plasma-modified nanofibers. (**C**) Storage stability of GOx immobilized on (**a**) unmodified and (**b**) air plasma-modified nanofibers. (**D**) SEM images of TiC CNFs. (**E**) TEM images of TiC CNFs; inset of (**E**) is the HRTEM image. (**F**) SEM image of GOx–TiC CNFs. (**A**–**C**) Reprinted with permission from [75]. Copyright 2016, Elsevier. (**D**–**F**) Reprinted with permission from [76]. Copyright 2018, Elsevier.

Glucose oxidase (GOx) is one of the main choices for real-time glucose monitoring owing to its high glucose selectivity. Nanofibers, due to their high specific surface area and porous structure, are excellent platforms for enzyme immobilization. The activity of the immobilized enzyme greatly affects the performance of the sensor. As implied in Figure 6A, the activity of GOx immobilized on the surface of PVA/malonic acid nanofibers with different plasma processing was quite different [75]. In addition, the reusability and storage stability of the plasma-treated fibers was significantly improved (Figure 6B,C). When designing the glucose sensor based on GOx, the nanofibers as the substrate have great influence on the performance of the sensor. Guo et al. produced CNFs with uniformly embedded TiC nanoparticles (Figure 6D,E) [76]. The robust adhesion of the composite nanofibers provides more abundant active sites for enzyme immobilization (Figure 6F). The GOx-TiC-CNFs biosensor could selectively detect glucose with a wide linear range (0.013–10.5 mM) and low detection limit (3.7 μM).

**Figure 7:**
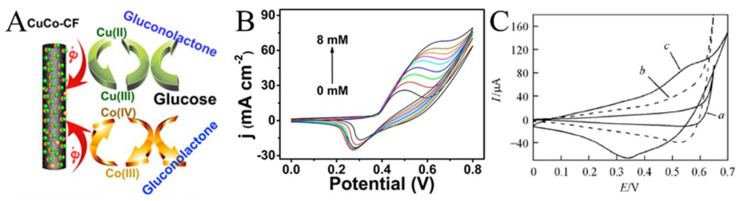
(**A**) Schematic diagram of the transition metal electrocatalysis detection of glucose. (**B**) CVs for Ni_2_P nanofibers in 0.1 M NaOH (pH 13) with the presence of varying glucose concentrations ranging from 0 to 8 mM. (**C**) CVs of (a) CuO-NFs, (b) CuO/CNFs, and (c) CuO/NiO-NFs film electrodes in 0.1 mol L^−1^ NaOH containing 0.6 mmol L^−1^ glucose at 50 mV s^−1^. (**A**) Reprinted with permission from [80]. Copyright 2016, American Chemical Society. (**B**) Reprinted with permission from [81]. Copyright 2016, American Chemical Society. (**C**) Reprinted with permission from [82]. Copyright 2013, WILEY-VCH.

Enzymes are unstable at certain temperatures and pH values. Therefore, non-enzyme sensors have been developed in which enzymes are generally replaced by metals or metal oxides. Non-enzyme sensors are more economical than enzymatic sensors and are less affected by the environment. Glucose is the most popular analyte. A variety of transition metals are used in non-enzyme electrochemical sensors for glucose. The mechanism diagram of the transition metal electrocatalytic detection of glucose is illustrated in Figure 7A. For example, Lu et al. reported a glassy carbon electrode (GCE) modified with CuO/Cu_2_O composite nanofibers for a glucose sensor [77]. CuO/Cu_2_O composite nanofibers had good electrocatalytic activity for glucose. The possible electrocatalytic processes of glucose can be explained as follows: The electrons produced by the oxidation of Cu^2+^ to Cu^3+^ form an oxidation peak in the cyclic voltammograms. After that, Cu oxidizes glucose to gluconolactone, which further increases the oxidation current [78,79,80]. In a specific range of glucose concentration, the oxidation peak increases linearly with the increase in glucose concentration. This property enables the determination of glucose concentration by the magnitude of the oxidation current (Figure 7B) [81]. Increased conductivity can accelerate electron transfer and improve the performance of the sensors. CNFs have excellent mechanical and electrical properties and can be simply obtained using carbonized electrospun polymer nanofibers, making them suitable as carriers for metal oxide nanoparticles. In addition, the synergistic effect of multiple components can also greatly enhance the electrocatalytic performance. The reason may be that the defects caused by the lattice mismatch of the two metal atoms lead to electron accumulation at the interface and the upward shift of the D band. Shi et al. prepared electrospun CuO nanofibers, CuO/CNFs, and CuO/NiO nanofibers as a glucose sensor, as shown in Figure 7C [82]. The electrocatalytic effect can be observed through the magnitude of the catalytic current and the position of the oxidation peak potential. Obviously, CuO/NiO nanofibers can produce higher current at lower potential.

There have been many reports on the application of electrospinning to non-enzyme electrocatalysts for the detection of glucose. Saravanan et al. prepared Co-Fe/PVdF-HFP by electrospinning and chemical reduction techniques for electrochemical glucose detection, in which the diffusion and adsorption of glucose in the expanded cavities and pores of the polymer nanofibers were accelerated to maximize the utilization efficiency of glucose [83]. Luo et al. fabricated a Pt-Au/polyurethane sensing patch through electrospinning, magnetron sputtering, and electrodeposition techniques for the determination of glucose in a neutral condition (pH 7.4) [84]. Li et al. synthesized hollow CuO/NiO nanoparticles with adjustable sizes by coaxial electrospinning and subsequent calcination [85]. The unique morphology and high specific surface area as well as the hetero-structural interface between CuO and NiO are conducive to improving the electrocatalytic performance, showing excellent electrocatalytic performance for glucose oxidation. They also prepared nano-Mn_3_O_4_/NiO-decorated CNFs by electrospinning and calcination [86]. The conductive network constructed by CNFs not only promotes the transfer of electrons but also provides a landing site for nanoparticles, thereby reducing the aggregation of nanoparticles and exposing more active sites. Kim et al. embedded MnO nanostructures with CNFs to significantly improve the detection performance of non-enzymatic amperometric glucose sensors [87]. Additionally, other nanofibers are also used in glucose biosensors, such as Ni_2_P/CNFs [88], PAN/PANI/CuO [89], CuSn/CNFs [90], and CuCo-P350 [91]. Table 1 summarizes several examples of non-enzyme electrochemical glucose sensors, including the detection limit, sensitivity, linear range, and oxidation potential.

### 4.2. H_2_O_2_ Sensors

In organisms, H_2_O_2_ is a signaling molecule that controls cell metabolism. In addition, it is also associated with some human diseases, such as cardiovascular diseases, Alzheimer’s Disease, and cancers [92]. Therefore, the detection of H_2_O_2_ is of great significance in medical applications.

**Figure 8:**
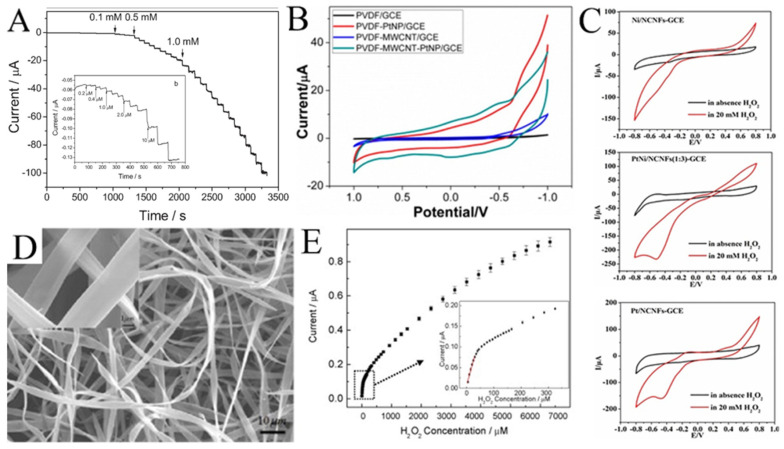
(**A**) Current–time responses of the Pd/CNFs-CPE upon successive injection of a specific concentration of H_2_O_2_ into N_2_-saturated PBS (0.1 M, pH 7.0); the inset (**b**) shows the performance of the Pd/CNFs-CPE in the amperometric detection of a low concentration of H_2_O_2_. Applied potential: −0.2 V. (**B**) CVs of GCEs modified with PVDF, PVDF-PtNP, PVDF-MWCNT, and PVDF-MWCNT-Pt hybrid nanofiber membranes. (**C**) CVs of Ni/CNFs-GCE, PtNi/CNFs-GCE, and Pt/CNFs-GCE in the absence and presence of H_2_O_2_ at 50 mV s^−1^. (**D**) Typical SEM images of Hb microbelts. (**E**) The corresponding calibration plot of amperometric response towards H_2_O_2_. Inset: enlarged drawing of the calibration plot for low H_2_O_2_ concentrations. (**A**) Reprinted with permission from [97]. Copyright 2008, WILEY-VCH. (**B**) Reprinted with permission from [98]. Copyright 2014, American Chemical Society. (**C**) Reprinted with permission from [99]. Copyright 2018, Elsevier. (**D**,**E**) Reprinted with permission from [100]. Copyright 2010, Elsevier.

Noble metal (such as Au [93], Ag [94], Pd [95], and Pt [96]) nanoparticles have excellent electrocatalytic activity with H_2_O_2_. For example, Huang et al. electrospun Pd nanoparticle-loaded CNFs to modify a carbon paste electrode (CPE) [97]. The modified CPE shows a wide linear range (0.2 μM to 20 mM) for H_2_O_2_ (Figure 8A). As shown in Figure 8B, Zhang et al. compared the cyclic voltammetry of H_2_O_2_ on PVDF, a multi-walled carbon nanotube (MWCNT), and Pt [98]. It was not difficult to see that the electrocatalytic performance of H_2_O_2_ mainly came from Pt. Without Pt, PVDF or MWCNT alone showed almost no current response to H_2_O_2_.

High cost limits the development of H_2_O_2_ sensors based on noble metals, so various transition metals/metal oxides are introduced to functionalize noble metals. As shown in Figure 8C, compared with Pt/CNFs and Ni/CNFs, the electrocatalytic performance of PtNi/CNFs for H_2_O_2_ is much better [99]. The PtNi/CNFs-modified GCE has a wide detection range from 0.05 μM to 8 mM with a low detection limit of 0.0375 μM for H_2_O_2_. Mohammadi et al. synthesized a Se/P@N-doped carbon nanobox (N-CNB)/CNFs for the electrochemical determination of H_2_O_2_ [21]. Due to the mesoporous structure, the open efficient diffusion channel for H_2_O_2_, the rapid mass/electron transfer, and the synergies between P, Se, and CNBs/CNFs, the synthesized Se/P@(N-CNB)/CNFs exhibited excellent electrocatalytic activity toward H_2_O_2_ oxidation. Hsueh et al. prepared IrO_2_@Ir NFs for the selective determination of H_2_O_2_ [86]. Qu et al. synthesized LaSrNiO NFs with a dual-phase structure and unique porous tubular nanofiber structure using electrospinning and high-temperature calcination techniques, significantly improving their redox performance for the electrochemical sensing of H_2_O_2_ [101]. Bi et al. modified the conductive polymer PEDOT:PSSLiTFSI-CoPc (PPLC) on nanofibers (PPLC/PU/PDMS) to develop a stretchable electrochemical sensor [102]. The electrode displayed good electrochemical sensing performance and stability under mechanical deformation. In addition, Ag@CuO [103], VCoO/C-750 [104], and Co-NC/CNF [105] are also used for H_2_O_2_ detection. Table 2 lists several examples of H_2_O_2_ sensors using electrospun metals/metal oxides nanofibers.

Interestingly, hemoglobin microbelts have been successfully electrospun for H_2_O_2_ sensing by Ding et al. (Figure 8D) [100]. In their work, hemoglobin is dissolved in 2,2,2-trifluoroethanol as the spinning solution for electrospinning hemoglobin microbelts, allowing for the sensitive detection of H_2_O_2_ at physiological pH (0.1 M pH 7.0 phosphate buffer). As shown in Figure 8E, this H_2_O_2_ sensor has a low detection limit (0.61 μM) and high stability due to its good biocompatibility and direct electron transfer capability.

### 4.3. Detection of Other Biomolecules

A variety of electrospun nanofibers have been developed for the detection of biomolecules, such as UA, DA, AA, protein, and amino acids. The level of these biomolecules in the body can reflect the health of the body and help doctors determine whether the body is suffering from certain diseases. For example, the concentration level of UA is one essential indicator for the diagnosis and the prognosis of some multifunctional disorders like gout, hypertension, and cardiovascular diseases [106]. DA is an important neurotransmitter, having strong influence on the central nervous, renal, cardiovascular, and hormonal systems [107].

**Figure 9:**
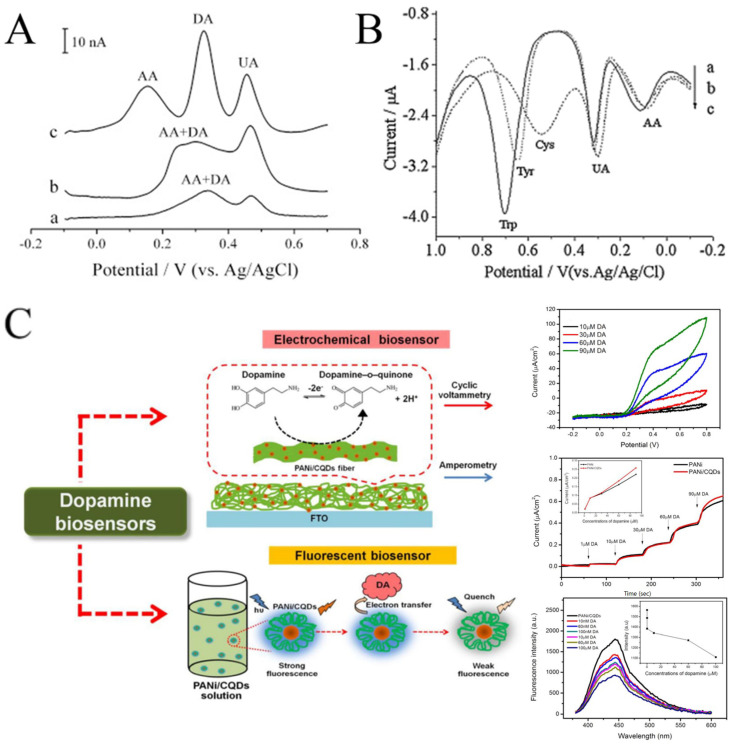
(**A**) DPVs at (a) CPE, (b) MWCNT-CPE, and (c) CNFs-CPE in 0.1 M PBS (pH 4.5) containing 2 μM DA, 6 μM AA, and 2 μM UA. DPV conditions: scan rate, 20 mV s^−1^; amplitude, 50 mV; pulse width, 100 ms; pulse period, 200 ms. (**B**) DPVs of CNFs-CPE in 0.1 M PBS (pH 7.0) containing 0.1 mM UA, 0.2 mM AA, and 0.2 mM Trp (a), 1 mM Cys (b), and 0.2 mM Tyr (c). DPV conditions: Scan rate, 6 mVs^−1^; amplitude, 50 mV; pulse width, 100 ms; pulse period, 200 ms. (**C**) Schematic diagram representing the developed electrochemical sensor and fluorescent sensor based on a PANi/CQDs composite. (**A**) Reprinted with permission from [108]. Copyright 2008, Elsevier. (**B**) Reprinted with permission from [109]. Copyright 2009, Elsevier. (**C**) Reprinted with permission from [112]. Copyright 2020, The Polymer Society.

CNFs have been widely used in electroanalysis due to their good dispersibility, wettability, conductivity, and biocompatibility. In some cases, the electrochemical performance of CNFs may be even better than that of CNTs (Figure 9A) [107,108]. CNFs can be easily obtained by carbonizing electrospun polymer nanofibers at high temperature. Tang et al. prepared electrospun CNFs-modified CPE to detect trace amounts of L-tryptophan (Trp), L-tyrosine (Tyr), and L-cysteine (Cys) [109]. The modified electrode showed a satisfactory linear range (0.1–118.5 μM for Trp, 0.2–109 μM for Tyr, and 0.15–63.8 μM for Cys). As implied in Figure 9B, in addition to the obvious current response to Trp, Tyr, and Cys, the CNFs-modified electrode also showed a response to UA and AA. The results show that CNFs can detect multiple biomolecules simultaneously by DPV. Recently, effort has been devoted to improving the electrocatalytic performance of CNFs, including the combination with MWCNTs and precious metals (such as Pd and Ag) [106,110,111].

An interesting example is the dopamine biosensor based on the electrospun PANi/carbon quantum dots (CQDs) composite nanofibers [112]. On one hand, CQDs have electrocatalytic activity with dopamine. On the other hand, the addition of dopamine can cause the fluorescence quenching of CQDs. Therefore, the electrospun PANi/CQDs composite nanofibers are capable of both electrochemical and fluorescent sensors for DA (Figure 9C). In another work, Samie et al. combined a novel RuO_2_-CeO_2_-AuNFs hybrid structure with graphite oxide and functionalized multiwalled carbon nanotubes for the simultaneous determination of serotonin, DA, and AA [113]. The proposed electrochemical sensor reduces overpotential and solves the problem of overlapping the oxidation peak potential. Yin et al. decorated nitrogen-doped electrospun CNFs with tightly packed Co_3_O_4_ nanoparticles with a high electroactive surface area and promoted electron transfer between the electrode surface and the target molecule by rapid electrodeposition [114]. The electrosensing platform showed excellent sensitivity and selectivity for DA. Veeralingam et al. demonstrated a high-performance field-effect transistor biosensor based on Al-functionalized β-Bi_2_O_3_ nanofibers for the highly selective and rapid detection of serotonin [115]. The biotransistor has good sensitivity, stability, and repeatability and a fast response time of 0.8 s.

Enzymes and antibodies could be immobilized on electrospun nanofibers for the specific identification and detection of biomolecules. For example, Mondal et al. covalently immobilized cholesterol esterase and cholesterol oxidase on electrospun TiO_2_ nanofibers with a high orientation for the detection of esterified cholesterol [116]. Atilgan et al. prepared dendrimer-modified montmorillonite-decorated poly-σ-caprolactone and chitosan-based nanofibers, which were conjugated with glutamate oxidase for the electrochemical determination of monosodium glutamate [117]. Proteins can be detected by immobilizing antibodies on electrospun nanofibers through the specific binding between antigens and antibodies. For example, Macwan et al. immobilized anti-C-reactive protein on electrospun PVA/CNT nanofibers for the detection of inflammatory marker C-reactive protein [118].

## 5. Challenges and Opportunities Facing Practical Application

**Figure 10:**
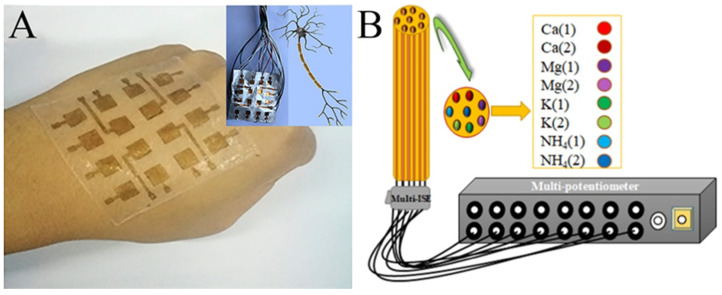
(**A**) Photograph of the e-skin based on a piezoelectric nanogenerator by electrospun PVDF nanofibers. The inset shows the demonstration of its analog to a neuron cell. (**B**) Schematic illustration of the multi-ion-selective electrode. (**A**) Reprinted with permission from [120]. Copyright 2018, American Chemical Society. (**B**) Reprinted with permission from [124]. Copyright 2020, Elsevier.

Although electrospinning technology has been deeply developed and its application has been extensively explored in many fields, there are still many issues left in both basic theory and practical application:

(i) Improvement of the theoretical model about jet: Although many models of jet movement in the electrostatic field have been established, these models are incomplete and are mostly half-empirical and half-theoretical [39,119,120]. In short, the theoretical model is still in its infancy.
(ii) Precise control of environmental factors: It has been widely reported that temperature and humidity have great influence on the morphology of electrospun nanofibers [42,121]. In the laboratory, the environmental factors of electrospinning are relatively easy to maintain. However, in order to realize the commercialization of electrospun nanofibers, large-scale production is inevitable. Under such circumstances, it is still a challenge to keep the environment stable.
(iii) Safety issues caused by solvent volatilization: A large amount of solvent volatilizes during the solidification of the jet. In large-scale production, the amount of solvent volatilization will become larger. Solvents widely used in electrospinning include DMF, DMSO, and sometimes acetone, which are toxic and may cause explosions.
(iv) Safety of nanofibers: Some research has suggested that nanofibers are likely to cause an inhalation hazard. For example, inhalation of a sufficient dose of asbestos nanofibers may cause mesothelioma [122]. At present, there are few studies on the inhalation safety of nanofibers. An in-depth study of this aspect is of great significance. Although electrospinning technology can protect our health, it can be more harmful if we ignore its own safety.
(v) Miniaturization of biosensors: With the advent of the 5G era, online medical treatment is available. People can enjoy medical services at home without the tedious procedures of offline medical treatment. Traditional testing methods usually require expensive testing equipment and complicated operations. Microscale biosensors would make online diagnosis easier. Several test papers and miniaturized instruments have been reported, but these fall far short of the need for online diagnoses [123,124]. The design of miniaturized biosensors may be a hotspot in the future.
(vi) The bionic device: Electrospun nanofiber mats mostly have excellent flexibility, which makes electrospinning technology promising in biomimetics. For example, PVDF, a piezoelectric material usually used for electrospinning, has been reported for the design of electronic skin (Figure 10A) [125,126,127]. There have also been a number of reports about electronic tongues, although they were only in the initial stage (Figure 10B) [128,129,130]. With the development of technology, it will be possible to integrate electronic tongues into real tongue sizes in the future. In addition, we can imagine that the PVDF nanofibers with piezoelectric properties could be used as not only electronic skin, but also electronic ears, an electronic throat, and even an electronic heart. On the software side, AI is developing rapidly. In this sense, bionic humans, the stuff of science fiction, may become a reality in the distant or even near future.

## 6. Conclusions

Electrospinning is a simple and efficient method for producing nanofibers. Electrospun nanofibers have large specific surface area, high porosity, and diversified structure. In addition, it is easy to functionalize the surface of electrospun nanofibers. These advantages make electrospinning technology have great potential in electrochemical sensors. In the past decades, a large number of studies have laid the foundation for the control of the structure and morphology of electrospun nanofibers, and many interesting composite nanofibers structures have been witnessed in practical applications of electrochemical sensing.

In this review, an overview was provided on the morphological and structural regulation of nanofibers by electrospinning technology as well as its application in electrochemical sensors. The factors affecting the morphology of the nanofibers and the strategies for producing nanofibers with special morphology were discussed. Generally, the performance of nanofibers can be improved and their application can be expanded through the following strategies: (i) nanofiber diameter; (ii) special morphology (hollow, core-sheath, porous and bead-like); (iii) functionalization of surfaces; (iv) metallization of nanofibers; and (v) carbonization to form CNFs. The working principles and advantages of various nanofibers obtained by these strategies in the detection of different substances were also discussed in this review.

In summary, many achievements have been made in both the theory and application of electrospinning technology in recent decades. Electrospinning has been involved in many fields, such as energy storage, tissue engineering, environmental protection, smart textiles, electronic devices, and physical and chemical sensors. In particular, the current research on electrospinning has a tendency of miniaturization, simplification, and economization, making it possible for the technique to play an important role in intelligent medical treatment. However, there are still many issues. For example, process and safety problems still exist in the large-scale production of electrospinning. In addition, it is necessary to establish a complete evaluation system for the safety of electrospun nanofibers.

## Figures and Tables

**Table 1:** Electrospun nanofibers for non-enzyme glucose electrochemical biosensors.

Catalyst	LOD (μM)	Sensitivity (μA mM^−1^ cm^−2^)	Linear Range (μM)	Potential (V)	Ref.
Co-Fe/PVdF-HFP	0.65	375.01	1–8000	0.53	[83]
TiO_2_/Cu_2_O/CuO CNFs	0.25	2074.7	0–2000	0.55	[80]
Pt-Au/polyurethane	14.77	203.13	0.1–50	N/R	[84]
CuO/NiO NFs	14.77	1324.17	1–1000	0.6	[85]
Mn_3_O_4_/NiO/CNFs	0.73	386.84	5–3000	0.5	[86]
MnOx-CNFs	0.3	4080.6	0–9100	0.55	[87]
Ni_2_P/CNFs	0.25	1050	5–208	0.5	[88]
PAN/PANI/CuO	1.2	N/R	3–500	0.4	[89]
CuSn/CNFs	0.08	N/R	0.1–9000	0.55	[90]
CuCo-P350	2.92	2272	5–825	0.55	[91]

**Table 2:** Electrospun nanofibers for H_2_O_2_ analysis.

Catalyst	LOD (μM)	Sensitivity (μA mM^−1^ cm^−2^)	Linear Range (μM)	Potential (V)	Ref.
Se/P@CNBs/CNFs	58	171.1	200–1800	NR	[21]
IrO_2_@Ir NFs	0.16	289	0.1–1000	−0.4	[86]
PtNi/CNFs	0.0375	248.5	0.05–8000	−0.1	[99]
LaSrNiO NFs	0.018	1667.9	1–7000	0.2	[101]
PPLC/PU/PDMS	0.2	0.0406	0.5–50	+0.55	[102]
Ag@CuO	0.01	1982.14	0.05–100	+0.6	[103]
VCoO/C-750	0.44	N/R	0–8000	N/R	[104]
Co-NC/CNF	10	300	1–6000	−0.5	[105]

## Data Availability

Not applicable.
